# DRESS with delayed onset acute interstitial nephritis and profound refractory eosinophilia secondary to Vancomycin

**DOI:** 10.1186/1710-1492-7-16

**Published:** 2011-10-03

**Authors:** Paloma O'Meara, Rozita Borici-Mazi, A Ross Morton, Anne K Ellis

**Affiliations:** 1 Department of Medicine, Queen's University, Kingston, Ontario, Canada; 2 Department of Biomedical and Molecular Sciences, Queen's University, Kingston, Ontario, Canada

## Abstract

**Background:**

Drug Reaction with Eosinophilia and Systemic Symptoms (DRESS) is a relatively rare clinical entity; even more so in response to vancomycin.

**Methods:**

Case report.

**Results:**

We present a severe case of vancomycin-induced DRESS syndrome, which on presentation included only skin, hematological and mild liver involvement. The patient further developed severe acute interstitial nephritis, eosinophilic pneumonitis, central nervous system (CNS) involvement and worsening hematological abnormalities despite immediate discontinuation of vancomycin and parenteral corticosteroids. High-dose corticosteroids for a prolonged period were necessary and tapering of steroids a challenge due to rebound-eosinophilia and skin involvement.

**Conclusion:**

Patients with DRESS who are relatively resistant to corticosteroids with delayed onset of certain organ involvement should be treated with a more prolonged corticosteroid tapering schedule. Vancomycin is increasingly being recognized as a culprit agent in this syndrome.

## Introduction

We present a case of severe Drug Reaction with Eosinophilia and Systemic Symptoms (DRESS) [1] syndrome secondary to vancomycin, with associated multiorgan dysfunction. The relatively high mortality of this syndrome warrants prompt recognition and elimination of the culprit drug and often treatment with high-dose corticosteroids.

## Case Report

A 66 year-old male presented to the emergency department (ED) with a one-week history of progressive pruritic erythematous rash, dry cough and two days of episodic high fevers. He had suffered a fall 12 weeks prior that had resulted in a pelvic fracture requiring an open-reduction internal fixation, which subsequently became infected with methicillin-resistant *Staphylococcus aureus *(MRSA) and treatment with intravenous vancomycin was initiated. After four weeks of vancomycin therapy he developed a rash. This was initially thought to be due to a red-man syndrome variant. Infusion rates were slowed, and premedication with diphenhydramine was initiated, but the rash worsened, with the subsequent development of episodic daily fevers, documented to be as high as 40°C.

His past medical history was significant for heterozygous hemochromatosis, a remote splenectomy secondary to traumatic rupture, and non-anaphylactic adverse reactions to penicillin and sulfa antibiotics. He had no history of reactive airway disease and had no travel history, living in an Ontario city.

**Figure 1:**
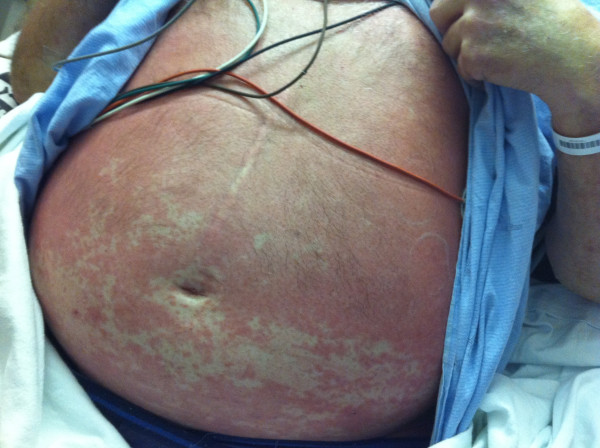
**Patient's rash on presentation**.

In the ED he was hemodynamically stable with a normal mental status. He had a severe erythematous macular rash involving his face and trunk (see Figure 1), and some facial edema. He had no mucous membrane involvement. He had palpable bilateral cervical and left axillary lymphadenopathy. Cardiorespiratory examination was normal, and his abdominal exam revealed central obesity and a scar from his remote splenectomy. Initial laboratory investigations are shown in Table 1. A computed tomography (CT) scan of his pelvis showed no abscess formation. A skin biopsy of the initial rash showed a mild perivascular lymphocytic infiltrate, consistent with a drug reaction.

**Table 1:** Laboratory Investigations (Initial)

**Parameter**	**Value**	**Reference**	**(Units)**	**Parameter**	**Value**	**Reference**	**(Units)**
	
Creatinine	**120**	50-100	umol/L	WBC	**30.2**	4.0-10.5	x10^9/L
	
Urea	**6.5**	3.0-6.5	umol/L	Eosinophils	**3.62**	0.0-0.4	x10^9/L
	
Sodium	**126**	135-145	mmol/L	Neutrophils	**19.34**	2.00-7.50	x10^9/L
	
Potassium	3.8	3.5-5.0	mmol/L	Lymphocytes	2.11	1.5-4.00	x10^9/L
	
Chloride	**93**	98-107	mmol/L	Hb	**114**	140-170	g/L
	
Glucose	5.0	3.5-11.1	mmol/L	Platelets	**604**	150-400	x10^9/L
	
Total Protein	**49**	60-80	g/L	ESR	20	< 30	mm/hr
	
Albumin	**21**	35-50	g/L	CRP	**13.4**	< 3.0	mg/L
	
INR	**1.3**	0.9-1.2		C3	1.22	0.80-1.8	g/L
	
Bilirubin	14	0-17	umol/L	C4	0.27	0.13-0.40	g/L
	
GGT	**354**	8-61	U/L	IgA	3.04	0.80-4.50	g/L
	
Alk Phos	**708**	56-119	U/L	IgD		< 140	mg/L
	
AST	**163**	12-45	U/L	IgE	70	< 129	IU/mL
	
ALT	**144**	7-40	U/L	IgG	13.02	6.00-16.00	g/L
	
CK	17	55-197	U/L	IgM	0.33	0.40-3.00	g/L
	
LDH	196	94-250	U/L				
	
Lactate	**3.1**	0.5-2.2	mmol/L	c-ANCA	Negative		
	
Blood cultures	Negative			p-ANCA	Negative		
	
Hepatitis B&C	Negative			ANA	Negative		
	
EBV serology	past exposure			ENA	Negative		
	
Stool O&P	Negative			SPEP	Negative		
	
Strongyloidiasis serology	Negative			UPEP	Negative		

**Figure 2:**
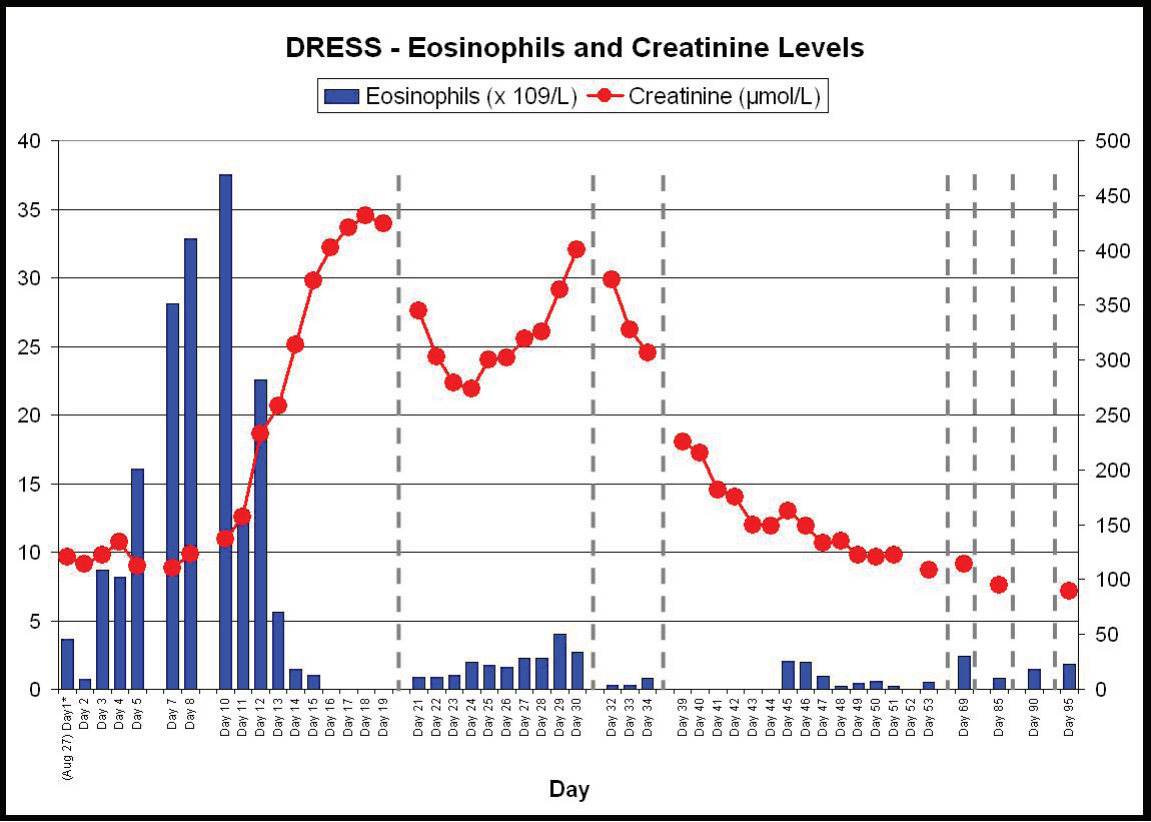
**Graph illustrating the eosinophil and creatinine trend over the course of the first three months following presentation**.

**Figure 3:**
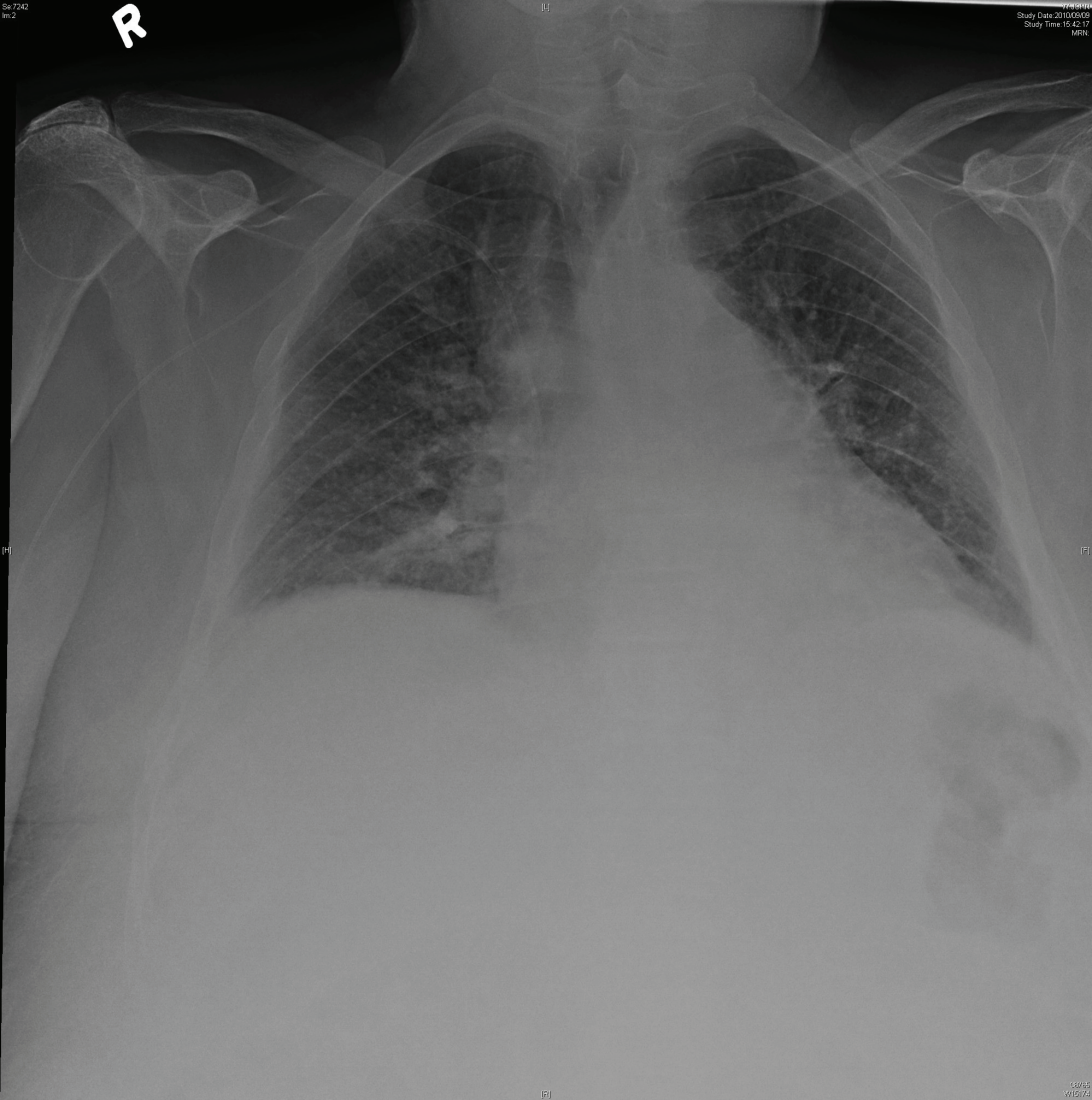
**Chest X-ray showing an interstitial pattern suggestive of eosinophilic pneumonitis in this patient**.

Our initial working diagnosis was Drug Reaction with Eosinophilia and Systemic Symptoms (DRESS) secondary to vancomycin, which was immediately discontinued. Initially, his liver enzymes and eosinophilia appeared to improve spontaneously, thus management was mainly supportive with topical hydrocortisone and oral H1 and H2 receptor antagonists (cetirizine 20 mg PO BID and ranitidine 150 mg PO BID). On the third day of admission, however, his white blood cell (WBC) and eosinophil counts increased and continued to rise (see Figure 2) leading to the initiation of systemic corticosteroids. He was initially given hydrocortisone 40 mg IV every 8 hours for 2 days followed by prednisone 60 mg PO daily. Despite this treatment, his eosinophil count continued to rise and peaked on Day 10 (see Figure 2). He became dyspneic with diffuse wheezing on exam, requiring repeated bronchodilator treatments. The chest x-ray was repeated and a CT chest performed. Chest imaging revealed a diffuse interstitial pattern that was suggestive of eosinophilic pneumonitis (Figure 3). There was no evidence clinically or on echocardiography of heart failure. While initially his renal function was close to normal, starting on Day 8 it began to worsen (see Figure 2), peaking on Day 18 at 432 micromol/L despite aggressive fluid administration and good urine output. A renal ultrasound was normal, but microscopic urinalysis revealed persistent WBC casts consistent with worsening interstitial nephritis as an explanation for the acute renal failure. During this time, he became delirious, with no causative abnormalities found on neurological examination, laboratory investigations or CT head, indicating the likely cause to be his underlying DRESS. High dose methylprednisolone 125 mg IV q8 hourly was initiated, and his renal function slowly improved, negating the need for a renal biopsy in the opinion of the consultant nephrologist. His wheezing and mental status also improved with the subsequent reduction in his WBC and eosinophil counts. Peripheral blood flow cytometry did reveal an atypical plasmacytoid population, but serum and urinary protein electrophoresis were negative for a monoclonal protein.

Our initially broad differential diagnosis was narrowed as our patient's clinical presentation developed. Red man syndrome, an infusion and dose-related mast cell degranulation in reaction to impurities found in vancomycin, was excluded early upon hospital presentation [2]. The fact that his rash persisted despite vancomycin discontinuation, the multisystem nature of his presentation and the moderate eosinophilia was suspicious for a more serious process. Churg-Strauss Syndrome (CSS) was also considered, but our patient had no history of asthma or any bronchodilator or corticosteroid use in the past, and therefore it was lower on our differential. Nonetheless, anti-neutrophil cytoplasmic antibodies (ANCAs), a test with only about 40% sensitivity for CSS, was sent and found to be negative [3]. This was sent 8 days after initiation of corticosteroids, which may have decreased the test's sensitivity. However the predictive value of a negative ANCA in the face of our low clinical probability was sufficient to exclude this diagnosis. CSS, often described as having three phases, the asthmatic, the hypereosinophilic and the vasculitic phases, is almost always preceded by a usually escalating asthmatic phase that can last up to years and is rarely subtle [3]. Seldom, asthma can be a late feature. However, we have followed our patient over time, and complete tapering of corticosteroid left him with no residual respiratory symptoms, making CSS highly unlikely.

Microscopic stool examination for ova and parasites was negative as was *Stongyloides stercoralis *serology. No further parasitic work-up was completed as the patient had no risk factors or gastrointestinal symptoms. Severe systemic bacterial infections, with the exception of scarlet fever, cause eosinopenia [4], and at the time of admission our patient had in fact been recovering from an MRSA bacteremia. Genetic testing for hypereosinophilic syndromes (HESs) with FIP1L1 and PDGFRA were negative, and although these are positive in only about 30% of patients with HESs [5], a secondary cause of hypereosinophilia was apparent and thus helped exclude this diagnosis with more certainty.

On day 39 from initial presentation, our patient returned to hospital with a methicillin-sensitive *Staphylococcus aureus *(MSSA) bacteremia and severe left shoulder pain. He was admitted under orthopedic surgery for exploration and debridement of both the shoulder and pelvis as well as removal of the pelvic hardware. Evidence of osteomyelitis in the pelvic bone was found during the surgery. He remains on cefazolin and is being followed by the infectious diseases service. Meanwhile, a taper of his prednisone was attempted around day 50 over a two week period. Unfortunately, his rash and eosinophilia returned shortly after its discontinuation, requiring re-initiation and a slower tapering attempt. This was finally successful more than four months after initial presentation. He is currently managed with an oral H1 receptor antagonist alone, and his peripheral eosinophil count has remained suppressed.

## Discussion

Drug Reaction with Eosinophilia and Systemic Symptoms (DRESS) is an idiosyncratic hypersensitivity response characterized by a maculopapular erythematous eruption that typically develops 2-6 weeks following initiation of the culprit drug. The typical findings include fever, lymphadenopathy, multisystem organ failure and eosinophilia or atypical lymphocytosis. The term DRESS was coined in 1996 by Bocquet *et al*., in an attempt to unify the many names given to different drug reactions thought to have a common pathophysiological mechanism [6]. It has been postulated that concomitant infection with herpes-simplex virus-6 (HSV-6) predisposes to development of DRESS [7] and recently suggested as a diagnostic requirement [8]. Multi-organ failure often presents in a stepwise fashion despite discontinuation of the culprit drug. The affected organs include, in order of frequency, the skin, liver, kidneys, lungs, heart, and more rarely CNS, thyroid, pancreas, colon, muscles and serosa. The most common drugs that cause DRESS are anti-epileptics, the first described being phenytoin in 1939 [9]. Nine cases of vancomycin-induced DRESS syndrome have been described so far in the English literature [10-18]. As well, a tenth case, although not labeled as such, fulfills the criteria of DRESS [19]. None of the described cases appear to have been as severe as what was observed in our case, and our patient was initially refractory to corticosteroids with a late onset to his acute kidney injury from interstitial nephritis.

Identifying patients with this syndrome is important, as mortality approaches 10% [1]. Treatment includes strict discontinuation of the culprit drug(s). Also, probability tools exist to help identify the most likely agents [20]. Supportive care with symptomatic treatment using H1 and H2 receptor antagonists and topical steroid treatment may be sufficient for some. It is recommended to start systemic corticosteroids when internal organ involvement is present [21]. Despite the lack of randomized controlled trials comparing supportive care alone to systemic steroids in the treatment of DRESS, experience has dictated their use and they are recommended by experts [15]. The use of systemic corticosteroids is further supported by observations of clinical worsening with early tapering of the same [22]. Occasionally, additional immunosuppressive therapy is necessary and has been observed to improve organ function [8].

## Conclusion

We present a case of a patient with a relatively severe DRESS syndrome secondary to vancomycin with multiple organ systems affected, including skin, hematological, liver, lung, brain and kidneys in a stepwise fashion. Onset of renal injury from acute interstitial nephritis was delayed and the response to standard doses of parenteral corticosteroids insufficient despite initial spontaneous improvements with the discontinuation of the offending drug. Additionally, skin and hematological abnormalities recurred once corticosteroids were tapered. Patients with DRESS who are relatively resistant to corticosteroids with delayed onset of certain organ involvement should be treated with a more prolonged corticosteroid tapering schedule. Vancomycin is increasingly being recognized as a culprit agent in this syndrome.

## Consent Statement

Written informed consent was obtained from the patient for publication of this case report and accompanying images. A copy of the written consent is available for review by the Editor-in-Chief of this journal.

## Competing interests

The authors declare that they have no competing interests.

## Authors' contributions

PO: Involved in care of patient, Literature review, Created initial drafts of manuscript and table, completed first round of revisions following reviewer feedback. RBM: Involved in care of patient, review/revisions to and approval of manuscript final draft. ARM: Involved in care of patient, review/revisions to and approval of manuscript final draft.

AKE: Involved in care of patient, critical review and revisions to manuscript prior to submission and post-reviewer feedback; created Figure 2; approval of manuscript final draft.

All authors read and approved the final manuscript.
